# One-Shot SERRS Detection of Iron and Acidity in Aqueous Systems

**DOI:** 10.1021/acsami.5c22602

**Published:** 2026-01-14

**Authors:** Irene Calderón González, Robbert Schuett, Gerwin Chilla, Florian Schulz, Zhiming Wang, Wolfgang J. Parak, Ramon A. Alvarez-Puebla

**Affiliations:** † Department of Physical and Inorganic Chemistry, 16777 Universitat Rovira i Virgili, 43007 Tarragona, Spain; ‡ Fachbereich Physik, Universität Hamburg, 20148 Hamburg, Germany; § School of Physics, 12599 University of Electronic Science and Technology of China, Chengdu 611731, China; ∥ Shimmer Center, Tianfu Jiangxi Laboratory, Chengdu 641419, People’s Republic of China; ⊥ ICREA − Institució Catalana de Recerca i Estudis Avançats, 08010 Barcelona, Spain

**Keywords:** SERRS, ion sensing, chemosensors. multiplexing

## Abstract

Optical strategies for ion sensing hold promise for portable and in situ analysis, yet their reliability is frequently limited by pH-dependent interferences that alter metal–ligand interactions. Herein, we present a multiprobe surface-enhanced resonance Raman scattering (SERRS) platform that enables simultaneous quantification of Fe­(II) and direct in situ pH readout in aqueous media. The sensor is constructed from polystyrene beads (PS) densely coated with silver nanoparticles and functionalized with two probes: phenanthroline (Phen), a metal-selective dye, hydrophobically entrapped within a cetrimonium bromide (CTAB) bilayer to retain its Fe­(II) binding activity, and 4-mercaptobenzoic acid (MBA), covalently anchored to the silver surface to provide a reliable pH response. Resonant excitation at 532 nm maximizes SERRS sensitivity, yielding Fe­(II) detection down to 30 ppb with strong selectivity against competing metal ions. Crucially, the MBA readout decouples pH effects from Fe­(II) quantification, avoiding false results at alkaline pH where iron hydroxide precipitates dominate. This dual-sensing strategy provides a robust concept to overcome pH interference in optical ion sensing and paves the way for portable, time-resolved monitoring of metal ions in biological and environmental systems.

## Graphical abstract

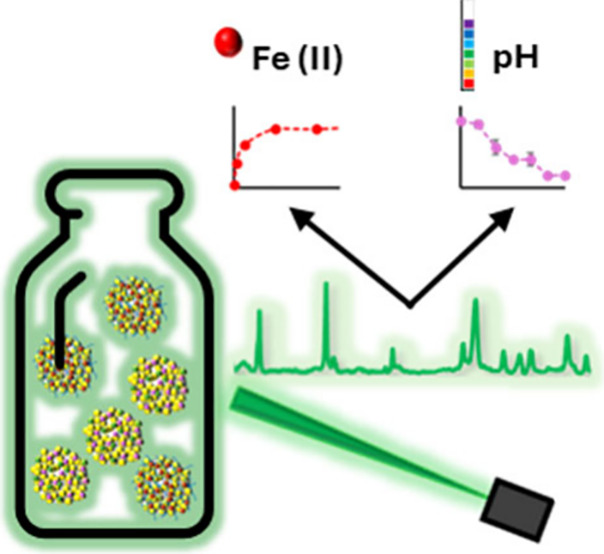

## 1 Introduction

Metal ions play essential roles both in the environment and in living organisms.^1^ Some, such as Na^+^, K^+^, or Ca^2+^ form the basis of cellular communication. Others, even at trace levels, are vital cofactors in enzymatic catalysis, electron transfer, and regulatory processes, with Fe­(II) in particular being central to redox chemistry, respiration, and DNA synthesis.^2,3^ However, when present in excess or in inappropriate chemical forms, many transition metal ions become toxic, promoting oxidative stress, protein misfolding, and organ damage in biological systems, as well as ecosystem imbalance in natural waters.^4^ For instance, the accumulation of Fe, Cu, Zn, or Co can disrupt aquatic life and contaminate drinking water supplies, while iron deficiency or overload in humans is directly linked to severe pathological conditions.^5^ This effect is used as one route to develop anticancer drugs leading to controlled cell death triggers by specific molecular pathways due to overdoses of Fe (i.e., ferroptosis)^6,7^ or Cu (i.e., cuproptosis).^8^ Metal ions are also of importance in the context of inorganic nanoparticles used toward biomedical applications.^9^

The dual role of metal ions, essential at controlled concentrations but harmful if dysregulated, has spurred growing interest in the development of sensitive and selective methods for metal ion detection.^10,11^ Several analytical techniques are available, including ion-selective electrodes, atomic absorption/emission spectroscopy (AAS/AES), anodic stripping voltammetry, and inductively coupled plasma mass spectrometry (ICP-MS).^12,13^ Although these methods provide low detection limits and enable the quantification of multiple ions, they often require extensive sample preparation, specialized personnel, and expensive, nonportable instrumentation. Consequently, they are typically restricted to laboratory-based analysis rather than time-resolved monitoring or on-site measurements. Some of them are also invasive, i.e. the sample is destroyed during measurement, such as in the case of ICP-MS, imposing further restriction on their application. As an alternative, optical sensors employing nanostructured substrates have emerged over the past decade and demonstrated great promise in ion sensing.^14,15^ Among them, surface-enhanced Raman scattering (SERS) is particularly attractive due to its high sensitivity and molecular specificity.^16,17^ Since SERS cannot directly detect atomic species, ion-selective dyes (chemosensors) are required.^13,15^ These dyes form complexes with specific metal ions and oxidation states,^18–20^ producing characteristic spectral changes that enable indirect ion quantification.^21^ However, a common limitation of many ion-selective dyes is the absence of functional groups that allow direct anchoring to plasmonic surfaces, often requiring chemical modifications that may compromise their binding ability. Embedding dyes in porous hosts, such as metal–organic frameworks (MOFs), has been explored to retain them in proximity to plasmonic substrates.^21–23^ While such approaches can enhance sensitivity and selectivity, the dyes may remain poorly exposed to target ions, reducing the efficiency of complex formation.

In addition to dye retention, one of the most critical challenges in optical ion sensing is interference from solution conditions. Competing ions can perturb the specificity of metal–ligand complexes, and, more importantly, pH strongly affects the stability of many complexes.^24–27^ Uncontrolled pH variations can therefore lead to misleading results, severely limiting the applicability of optical ion sensors. This limitation is particularly relevant for Fe­(II), as its aqueous speciation is strongly influenced by pH, with hydroxide precipitation occurring in alkaline conditions. Thus, strategies capable of simultaneously monitoring both Fe­(II) and pH are essential to achieve reliable quantification.

One solution lies in multiplexed measurements, i.e. measuring the ions of interest, as well as the ions which interfere with these measurements.^28,29^ An example has been shown for the detection of Na^+^ and K^+^ for responsive fluorophore, whose fluorescence also depends on pH apart from the Na^+^ and K^+^ concentration. By reading out the Na^+^ and K^+^ concentration and recording the pH of the solution in parallel, the interference of pH on the Na^+^ and K^+^ readout could be corrected based on calibration curves.^27^

Here, we address these challenges with a similar concept, but by using surface-enhanced resonance Raman scattering (SERRS)^30,31^ as readout. This was achieved by developing a dual SERRS platform based on polystyrene (PS) beads decorated with silver nanoparticles (AgNPs). Phenanthroline (Phen), a classical Fe­(II)-selective ion-selective dye,^32,33^ is hydrophobically entrapped within a cetrimonium bromide (CTAB) layer as a proof-of-concept strategy to preserve its complexation ability while remaining close to plasmonic hotspots. At the same time, 4-mercaptobenzoic acid (MBA), a well-established pH-selective dye,^34,35^ is covalently attached to the AgNPs, enabling independent and direct in situ monitoring of pH variations. Under green light excitation, resonance with the Fe–Phen charge-transfer transition ensures strong SERS enhancement, while the MBA band shift provides a direct pH readout. By combining these two complementary signals in a single nanostructured platform, we demonstrate simultaneous Fe­(II) quantification and pH monitoring in aqueous media under controlled conditions. This dual approach not only ensures reliable ion detection within acidic pH regimes, but also helps to circumvent the long-standing issue of pH interference in optical sensing, thereby providing a generalizable framework that can be extended to other metal ions through appropriate ion-selective dyes and adapted to more complex systems in future studies.

## 2 Results and Discussion

A straightforward and reproducible strategy was adopted to construct SERS-active substrates with strong enhancement, while maintaining synthetic simplicity. For this purpose, 300 nm diameter zwitterionic polystyrene (PS) beads functionalized with amine and carboxylate groups were chosen as underlying support particles to which the other components are attached, since these functionalities provide multiple binding sites for metallic NPs. Silver nanoparticles (AgNPs), prepared following a reported procedure,^36^ were then attached to the PS beads, taking advantage of their high affinity for both surface groups (Figure 1a). The AgNPs displayed an average core diameter of 15.7 ± 1.7 nm as measured by transmission electron microscopy (TEM), and a localized surface plasmon resonance (LSPR) centered at 405 nm (Figure 1b). Attachment of AgNPs to the PS beads induced a redshift in the LSPR (Figure 1c), confirming plasmon coupling between adjacent nanoparticles and the formation of SERS hotspots.^37^ Scanning electron microscopy (SEM) images (Figure 1c) further demonstrated a uniform coverage of AgNPs on the bead surface, confirming the successful fabrication of AgNP-decorated PS beads (PS@Ag).

**Figure 1:**
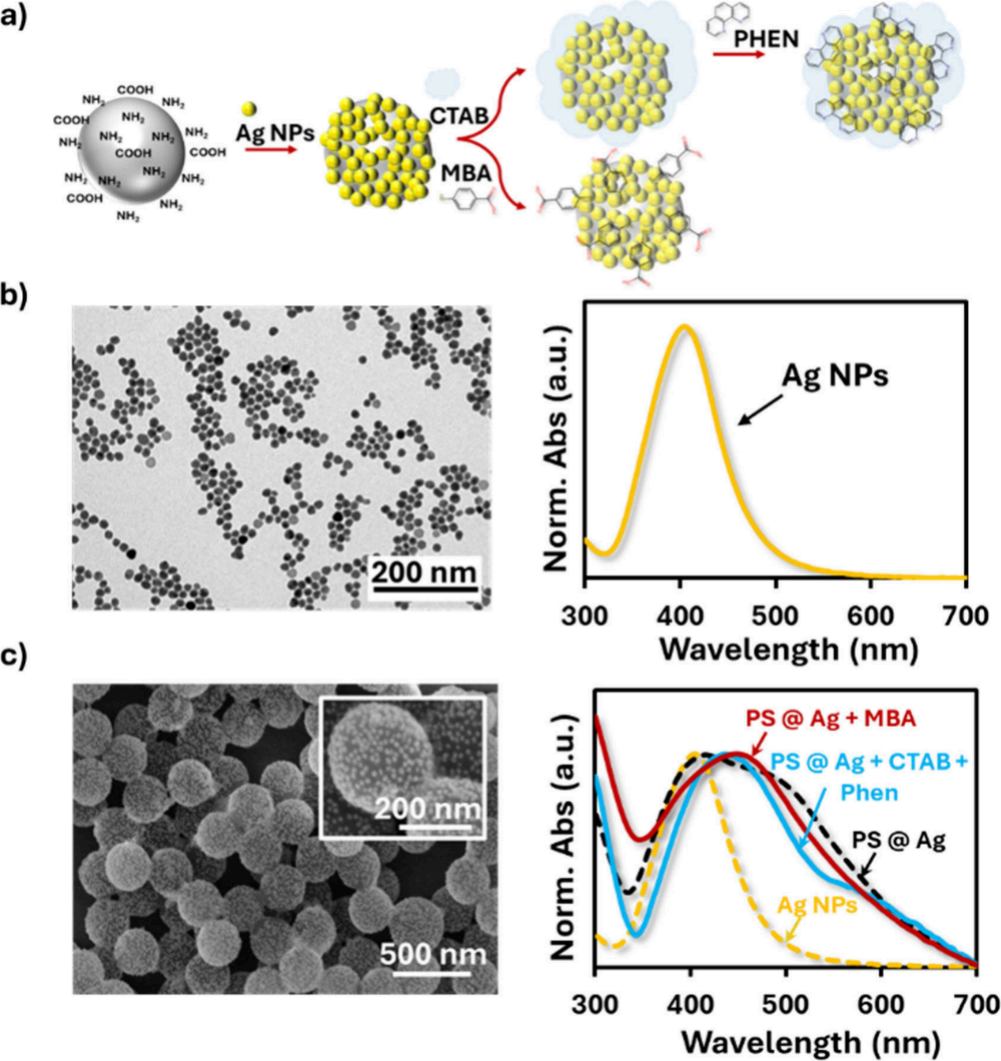
(a) Schematic representation of 300 nm zwitterionic polystyrene (PS) beads functionalized with amine and carboxylate groups and decorated with silver nanoparticles (AgNPs). (b) TEM image and normalized UV–vis absorption spectrum of citrate-capped AgNPs showing an average diameter of 15.7 ± 1.7 nm and a plasmon resonance centered at 405 nm. (c) SEM image and UV–vis absorption spectra of PS@Ag, PS@Ag functionalized with MBA, and PS@Ag coated with CTAB and Phen, highlighting the redshift of the LSPR upon AgNPs attachment and dye incorporation.

Phenantroline (Phen) was employed as an ion-selective dye to monitor Fe­(II) concentration due to its well-established ability to form the ferroin complex with octahedral coordination around Fe­(II).^32,33^ The electronic absorption spectra (Figure 2a) illustrate the characteristic features of this system. Free phenanthroline shows intense π→π* transitions in the UV region (∼270 nm) and a weaker n→π* band around 330 nm. Upon complexation with Fe­(II), an additional broad absorption line centered at ∼510 nm appears, assigned to a metal-to-ligand charge transfer (MLCT) transition. This MLCT band is responsible for the deep red color of ferroin.^38^ Notably, the spin-allowed d–d transitions expected for a low-spin d^6^ octahedral complex (from the ground state ^1^A_1g_ to excited states ^1^T_1g_ and ^1^T_2g_, according to the Tanabe–Sugano^39^) are Laporte-forbidden and therefore very weak.^40^ As a result, they are not observed in the UV–vis absorption spectra, which are dominated by the intense MLCT band.

**Figure 2:**
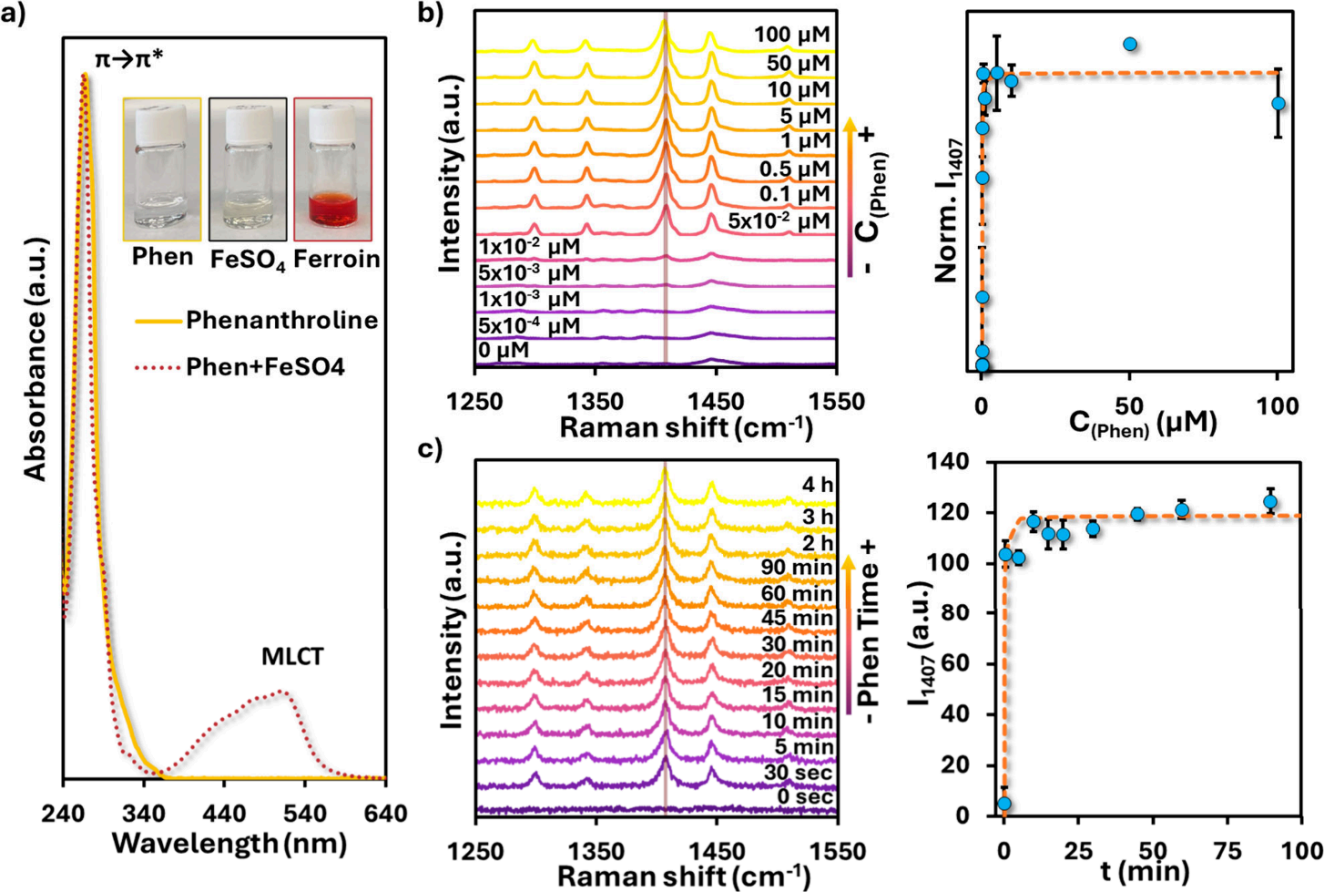
(a) UV–vis absorption spectra of phenanthroline and its Fe­(II) complex (ferroin). Phenanthroline shows intense π→π* and n→π* transitions in the UV region, while ferroin exhibits an additional broad band at ∼510 nm assigned to a metal-to-ligand charge transfer (MLCT). Insets: photographs of phenanthroline, FeSO_4_, and ferroin solutions, highlighting the characteristic red color of the MLCT band. (b) Concentration-dependent SERS spectra (i.e., intensity of the Raman) of phenanthroline at added concentration C_Phen_, incubation time *t* = 2 h, adsorbed onto PS@Ag + CTAB substrates (left), showing the growth of the 1407 cm^–1^ marker band (asymmetric in-plane ring stretching). The calibration plot (right; normalized intensity at 1407 cm^–1^ versus the phenanthroline concentration I_1407_(C_Phen_)) follows a Langmuir-type isotherm, with saturation reached at ∼5 μM. (c) Time-dependent SERS spectra of phenanthroline after addition of C_Phen_ = 1 mM to PS@Ag + CTAB at different incubation times (left). The intensity of the 1407 cm^–1^ band increases rapidly within the first 10 min and remains stable up to 4 h. The corresponding kinetic trace (right) follows a first-order model, confirming equilibrium between free and adsorbed phenanthroline.

Vibrational characterization of the Phen and its complex with iron, either free or on the plasmonic material, with different laser lines provides further insight (Figure S1). The Raman spectrum of free phenanthroline exhibits distinct ring stretching modes at 1345 cm^–1^ (in-phase C=C stretching), 1407 cm^–1^ (asymmetric in-plane stretching), 1600 cm^–1^ (out-of-phase in-plane stretching), and 1623 cm^–1^ (in-phase stretching).^41,42^ At 785 nm excitation, both phenanthroline and ferroin display weak Raman features, and their SERS spectra show only moderate enhancement on PS@Ag substrates. In contrast, excitation at 532 nm greatly increases the vibrational intensity ferroin due to the resonance with the MLCT band. The combination of resonance and plasmonic amplification produces sharp and intense SERRS spectra with dominant marker bands at 1345, 1407, 1600, and 1623 cm^–1^.

Phen was retained within the nanostructure through hydrophobic entrapment using a CTAB bilayer. While CTAB is typically employed as a stabilizer or reshaping agent for plasmonic nanoparticles, here it was used to generate a hydrophobic microenvironment around the PS@Ag surface. CTAB molecules possess both, hydrophilic and hydrophobic domains and interact electrostatically with the nanoparticle surface. At sufficiently high concentrations, they self-assemble into bilayers that produce a hydrophobic layer in close proximity to the metal surface while maintaining an overall hydrophilic character of the nanostructure.^43–45^ This approach ensured that Phen was localized near plasmonic hotspots without requiring chemical modification, thereby preserving its Fe­(II)-binding capability. The adsorption of phenanthroline into PS@Ag + CTAB was then evaluated (Figure 2b). The stacked spectra reveals that at very low Phen concentrations (<10^–8^ M), no detectable bands are present. The characteristic 1407 cm^–1^ marker band appears around 5 × 10^–8^ M, albeit with low signal-to-noise, and becomes progressively more defined with increasing concentration. Above 10^–8^ M, the 1407 cm^–1^ band and the associated ring stretching modes at 1345, 1600, and 1623 cm^–1^ are clearly resolved. At 5 μM, the signals reach a plateau, indicating that the adsorption sites available in the CTAB bilayer are saturated. The calibration plot (Figure 2b, right) shows that the normalized intensity of the 1407 cm^–1^ band follows a Langmuir-type isotherm, consistent with a monolayer sorption mechanism, where a finite number of binding sites are progressively filled until saturation. This behavior strongly supports the proposed entrapment mechanism, in which phenanthroline accumulates in the hydrophobic CTAB domains adjacent to the plasmonic surface. Control experiments performed using PS@Ag beads in the absence of CTAB showed no detectable Phen SERS signal after washing, confirming that Phen retention arises from CTAB-mediated hydrophobic entrapment rather than nonspecific adsorption to the Ag surface. The adsorption kinetics were further investigated by monitoring the 1407 cm^–1^ band after addition of 1 mM Phen (Figure 2c). The spectra show that the band emerges within seconds of mixing, increases steeply during the first 5–10 min, and gradually levels off, reaching equilibrium after ∼10 min. The kinetic trace (Figure 2c, right) fits a first-order model, indicating that the rate of adsorption is governed by the concentration of unoccupied sites rather than by diffusion. Importantly, the signal remained stable over extended periods (up to 4 h), demonstrating that the CTAB bilayer provides a robust and long-lived environment for phenanthroline entrapment.

The interaction between Fe­(II) and phenanthroline was further examined to validate the suitability of the system for quantitative sensing. The changes in the Raman spectra upon complexation are illustrated in Figures S2 and 3. In Figure S2, both the UV–vis absorption spectra (left) and the vibrational spectra (right) are shown for Phen with different Fe:Phen stoichiometric ratios (1:1, 2:1, 3:1). The absorption spectra are dominated by the π→π* and n→π* transitions of the ligand and by the metal-to-ligand charge transfer (MLCT) band of ferroin in the visible region. Importantly, the overall spectral profile remains essentially unchanged across different stoichiometries, confirming that the π* manifold of Phen is essentially preserved regardless of whether the Fe­(II) center coordinates one, two, or three ligands. Consequently, the general shape and position of the MLCT envelope is largely unchanged across [Fe­(phen)]^2+^, [Fe­(phen)_2_]^2+^, and [Fe­(phen)_3_]^2+^.^46–50^ This explains why the UV–vis absorption spectra of mono-, bis-, and tris-phenanthroline complexes appear nearly identical and why the Fe­(II) concentration can be quantified independently of the complex stoichiometry.

To enable quantification by Raman spectroscopy, the relative intensity changes of two neighboring marker bands were monitored: the increasing band at 1453 cm^–1^, in-plane stretching vibrations of the phenanthroline ring,^42,46,49,50^ and the decreasing band at 1446 cm^–1^, ring stretching modes (Figure 3a). Deconvolution of the overlapping bands (inset,Figure 3 **a**) enabled accurate tracking of intensity ratios (I_1453_/I_1446_), which increased systematically with Fe­(II) concentration. The resulting calibration curve followed a Langmuir-like behavior with a sharp rise at low concentrations, indicating a sorption-dominated complexation process between Fe­(II) and phenanthroline. A plateau was reached around 10 ppm Fe­(II), while the limit of detection (LOD) was estimated to be ∼ 30 ppb, highlighting the high sensitivity of the system. All experiments were conducted using a single, well-characterized batch of PS@Ag beads to ensure internal consistency. Multiple independently prepared samples were measured on different days, yielding reproducible intensity ratios and calibration trends. The limit of detection ranged between ∼27 and 54 ppb across independent measurements.

**Figure 3:**
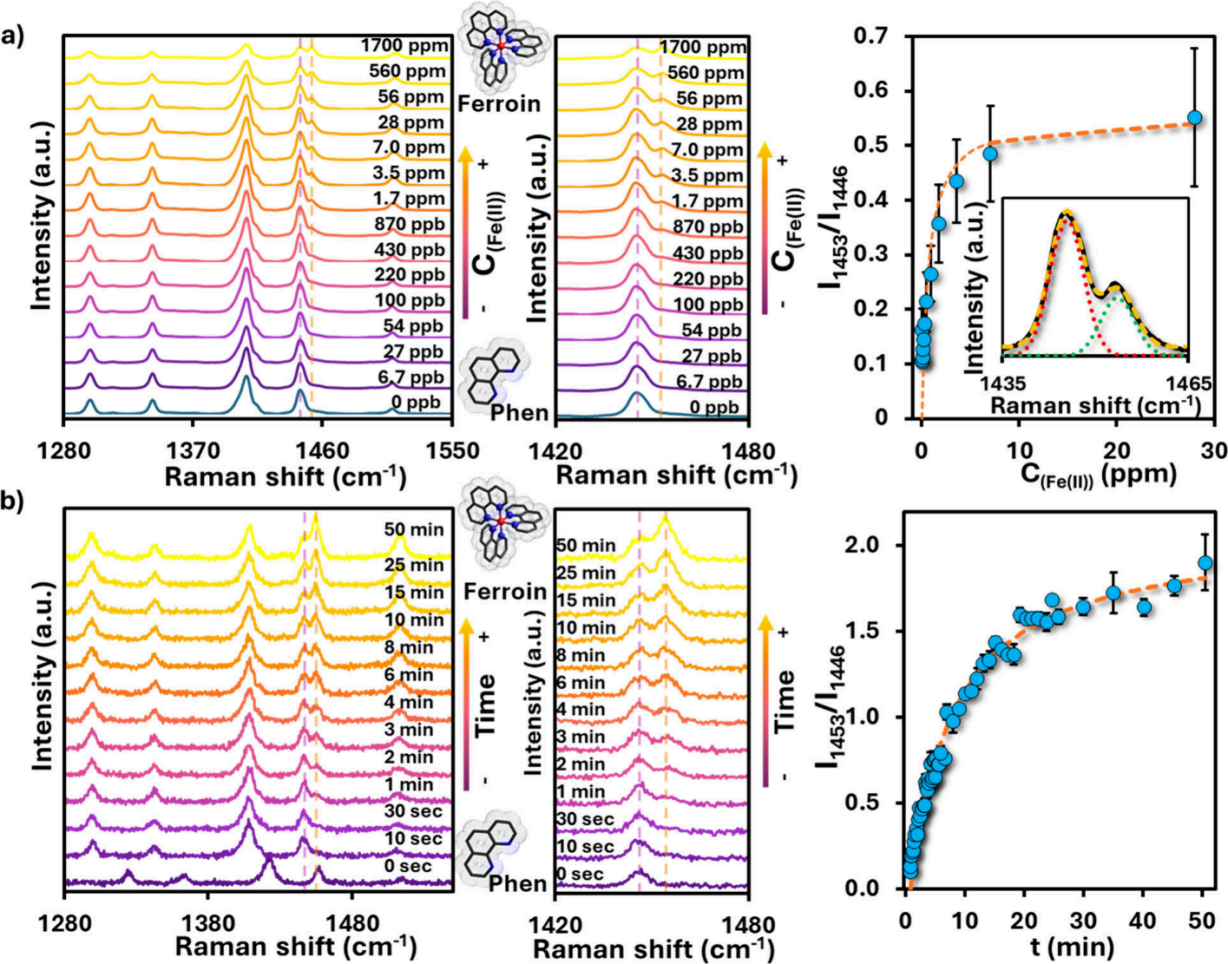
(a) SERRS spectra of PS@Ag + Phen after addition of increasing Fe­(II) concentrations C_Fe(II)_ (left; C_Phen_ = 10 μM; incubation time *t* = 3 h), highlighting the relative intensity changes of the 1453 cm^–1^ (in-plane ring stretching) and 1446 cm^–1^ (ring stretching) bands. Inset: deconvolution of overlapping peaks at 1440–1460 cm^–1^ used for ratiometric analysis. A plateau is reached at ∼10 ppm Fe­(II), with a limit of detection (LOD) of ∼30 ppb. Note, for diluted solutions 1 ppb corresponds to a mass concentration of 1 μg/L, given the molar mass of iron M­(Fe) = 55.84 g/mol to a molar concentration of 18 nmol/L. (b) Time-dependent SERS spectra after addition of Fe­(II) (left), showing a progressive increase with incubation time t of the 1453 cm^–1^ band and decrease of the 1446 cm^–1^ band. The kinetic trace of I_1453_/I_1446_ (right) follows a first-order model, reaching equilibrium after ∼20 min.

The time evolution of the same spectral features after Fe­(II) addition was also examined (Figure 3b). The stacked spectra demonstrate a progressive increase in the 1453 cm^–1^ band and a concurrent decrease in the 1446 cm^–1^ band, reaching equilibrium after approximately 20 min. The corresponding kinetic plot follows a first-order model, consistent with a process limited by the availability of uncomplexed phenanthroline sites rather than diffusion. Compared to the rapid physisorption of phenanthroline into CTAB bilayers (which equilibrates within ∼ 10 min, Figure 2c), the slower kinetics observed here reflect the additional structural reorganization required for Fe­(II) complexation. This confirms that the rate-limiting step is the formation of the ferroin coordination sphere rather than dye entrapment.

Since pH interference is a well-known challenge in optical ion sensing due to its effect on metal–ligand equilibria, the impact of pH on Fe­(II) quantification via SERRS was evaluated (Figure 4). The PS@Ag + CTAB + Phen structures exhibited a clear pH-dependent response to Fe­(II) concentration. Monitoring the same marker bands used previously to track complex formation (1453 cm^–1^ and 1446 cm^–1^, see Figure 3), the expected spectral changes—decrease of the 1446 cm^–1^ band with a concomitant increase of the 1453 cm^–1^ band—were only observed under acidic conditions (Figure 4a). At neutral and basic pH, however, the characteristic 1453 cm^–1^ band did not appear even at elevated Fe­(II) concentrations, indicating that complexation was strongly suppressed. The complete set of SERRS spectra for all pH values is provided in Figure S3. The calibration curves in Figure 4b further confirm this behavior. At acidic pH (pH 3 – 6), the intensity ratio I_1453_/I_1446_ increased with Fe­(II) concentration, following a Langmuir-type isotherm similar to that observed in Figure 3a. This demonstrates that reliable quantification of Fe­(II) is possible in acidic media. In contrast, at near-neutral and basic pH (pH 7–9), the ratio remained essentially constant and independent of Fe­(II) concentration, precluding quantitative analysis. The drastic decrease in Fe­(II) sensitivity at higher pH can be attributed to the formation of poorly soluble iron hydroxide species, which compete with phenanthroline for Fe­(II) coordination and limit ferroin formation.^51,52^ This interpretation is supported by visual inspection of the solutions (Figure 4b, insets): the characteristic red color of ferroin decreases in intensity with increasing pH, and visible precipitation is observed at pH 9. UV–vis study of the complex at different pH values confirms the absence of complex formation at basic pH conditions (Figure S4). Thus, it should be emphasized that reliable Fe^2+^ quantification is restricted to acidic conditions (pH 3–6), as Fe^2+^ hydrolysis and precipitation at higher pH fundamentally limit optical detection

**Figure 4:**
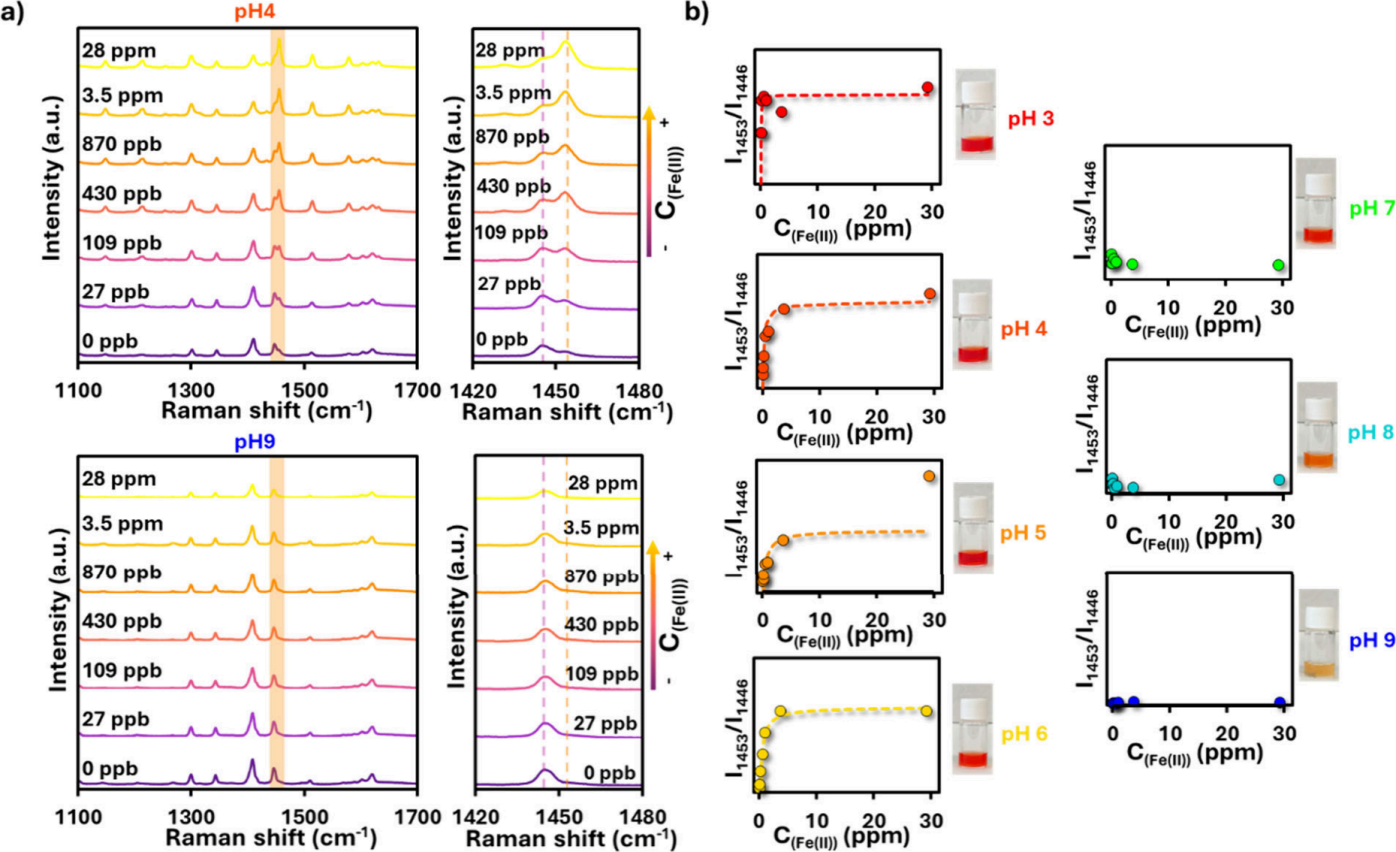
(a) SERRS spectra of PS@Ag + CTAB + Phen (*C*_Phen_ = 10 μM, *t* = 3 h; the same parameters are used in all following images) recorded at different Fe­(II) concentrations and pH values (examples shown for pH 4 and pH 9). The characteristic changes in the 1453 cm^–1^ (in-plane ring stretching) and 1446 cm^–1^ (ring stretching) bands are observed under acidic conditions, while at basic pH no spectral response is detected. (b) Calibration plots of the intensity ratio I_1453_/I_1446_ as a function of Fe­(II) concentration at different pH values. At acidic pH (3 – 6), the ratio follows a Langmuir-type response, enabling quantitative Fe­(II) detection. At neutral and basic pH (7 – 9), no response is observed due to precipitation of Fe­(OH)_2_ species competing with complex formation. Insets show photographs of solutions, where the intensity of the characteristic red ferroin color decreases with increasing pH, disappearing completely at pH 9.

To enable simultaneous monitoring of Fe­(II) concentration and pH, MBA, a widely used pH reporter molecule, was covalently bound to PS@Ag structures. Mixing colloidal solutions of the Fe^2+^ sensor (PS@Ag + CTAB + Phen) with the pH sensor (PS@Ag + MBA) produced combined SERRS spectra in which both reporters could be observed (Figure 5a). The complete set of SERRS spectra recorded at different Fe^2+^ concentrations and pH values is provided in Figure S5. Traditionally, the vibrational bands associated with the carbonyl (C = O) stretching mode at ∼1700 cm^–1^ and the carboxylate (COO^–^) symmetric stretching at ∼ 1400 cm^–1^ are chosen for pH sensing.^53^ However, in this hybrid system, these bands overlapped with Phen signals, preventing their reliable use. As shown in Figure 5b, no consistent trend in the intensity ratio I_1700_/I_1419_ was obtained across the investigated pH range. Instead, the ring stretching vibration at 1587 cm^–1^ was identified as a robust pH marker that is free from interference by Phen (Figure S6). As reported previously,^34,54^ protonation and deprotonation of the carboxyl group in MBA affects the electronic distribution of the aromatic ring, resulting in a systematic shift of the 1587 cm^–1^ band. Using spectral deconvolution, we determined that this band shifts linearly with pH from 3 to 9 (Figure 5c), providing a reliable calibration curve. Importantly, this pH-dependent spectral shift was unaffected by the Fe^2+^ concentration (Figure S7), confirming that MBA allows for independent pH readout in the multiplexed sensing platform. Thus, while phenanthroline provides selective Fe^2+^ detection through the ratio of the 1453/1446 cm^–1^ bands (Figures 3–4), MBA enables simultaneous monitoring of pH through the position of the 1587 cm^–1^ band. Together, these results establish a dual-sensing approach capable of quantifying Fe^2+^ in aqueous environments while correcting for pH interference.

**Figure 5:**
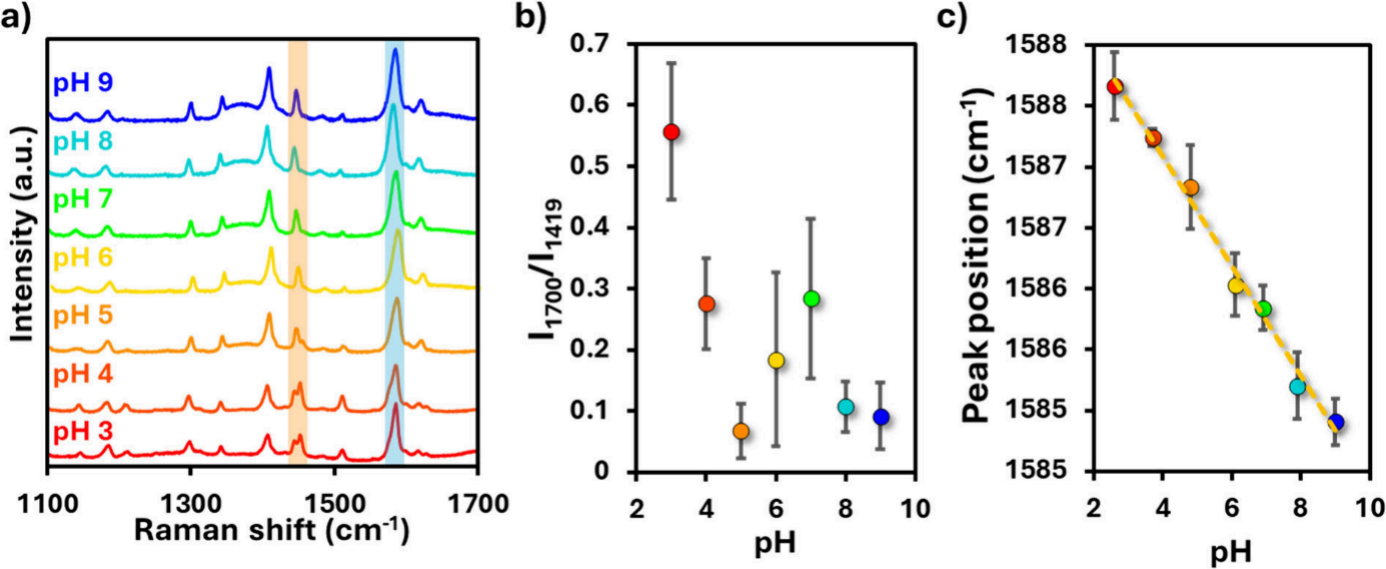
(a) Combined SERRS spectra of PS@Ag + CTAB + Phen (Fe^2+^ reporter) and PS@Ag + MBA (pH reporter) at different pH values. (b) Intensity ratio *I*_1700_/*I*_1419_, typically used for pH readout via MBA, shows no clear pH dependence due to overlap with phenanthroline signals. (c) Peak position of the MBA ring stretching band at ∼1587 cm^–1^ as a function of pH, determined by spectral deconvolution. A linear shift is observed across pH 3–9, enabling reliable pH quantification without interference from phenanthroline.

As shown in Figure 6a, Fe^2+^ quantification was simultaneously performed alongside the pH readout using the same spectral markers previously established (1453 cm^–1^ and 1446 cm^–1^, corresponding to ring stretching vibrations). The response closely mirrors the pH-dependent behavior described in Figures 4 and 5; at acidic pH, the 1453 cm^–1^ band increases in intensity while the 1446 cm^–1^ band decreases, enabling reliable quantification of Fe^2+^ concentrations. Importantly, no spectral overlap with the MBA reporter was observed, confirming that the Fe^2+^ readout remains uncompromised in the multiplexed sensing system (Figure S6). At basic pH, however, the Fe^2+^ response is completely suppressed, consistent with the precipitation of iron hydroxide species competing with phenanthroline complexation, as previously discussed (Figure 4). The corresponding calibration plots (Figure 6b) show that under acidic conditions the ratio I_1453_/I_1446_ increases with Fe^2+^ concentration, following a Langmuir-type model with a sharp initial rise indicative of high affinity binding. Concentrations as low as 30 ppb could be clearly detected, while saturation occurs at ∼5 ppm, consistent with the binding capacity of the system. At neutral and basic pH (pH 7–9), no usable Fe^2+^ signal is obtained. Nevertheless, the simultaneous pH readout provided by MBA prevents false positives or misinterpretation of the absence of Fe^2+^ signals.

**Figure 6:**
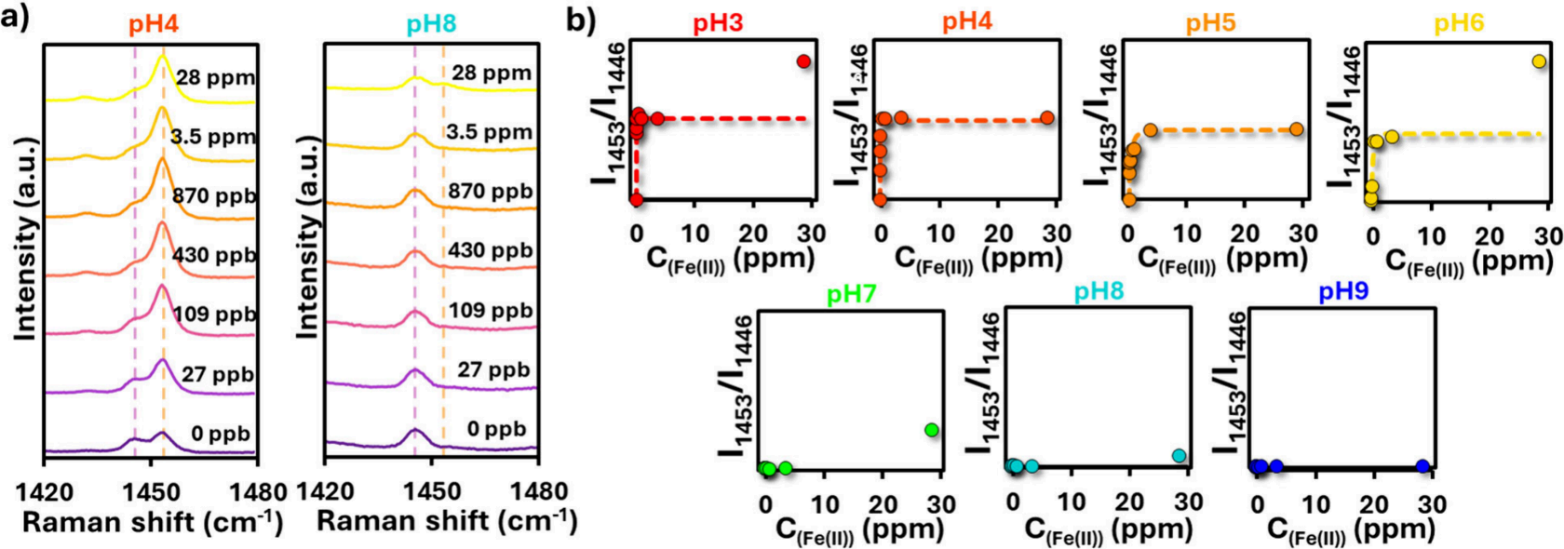
(a) SERRS spectra of PS@Ag + CTAB + Phen (Fe^2+^ reporter) in the presence of increasing Fe^2+^ concentrations at pH 4 (left) and pH 8 (right). Characteristic changes in the 1453 cm^–1^ (increase) and 1446 cm^–1^ (decrease) bands are observed only under acidic conditions, while no response is detected at basic pH due to the formation of insoluble iron hydroxides. (b) Calibration curves of the *I*_1453_/*I*_1446_ ratio as a function of Fe^2+^ concentration at different pH values. At acidic pH (3–6), the response follows a Langmuir-type trend with a detection limit of ∼30 ppb and saturation at ∼5 ppm. At neutral and basic pH (7–9), no measurable Fe^2+^ response is observed, but the simultaneous MBA-based pH readout prevents false quantification.

Another common source of interference in metal–ligand based sensors, besides pH effects, is the presence of competing transition metal ions.^55^ To assess the selectivity of the system, SERRS spectra were recorded in the presence of Fe^2+^, Co^2+^, Cu^2+^, and Zn^2+^ at acidic pH (Figure 7a). The characteristic spectral changes used for Fe^2+^ detection, namely the decrease of the 1446 cm^–1^ band and the concomitant increase of the 1453 cm^–1^ band, were only observed for Fe^2+^. In contrast, none of the other tested ions produced significant modifications in these bands, even at concentrations comparable to or higher than those of Fe^2+^. This high selectivity is further illustrated in Figure 7b, where the ratio *I*_1453_/*I*_1446_ is plotted as a function of ion concentration. While Fe^2+^ produces the expected Langmuir-type response with saturation at a few ppm (as in previous experiments, Figures 3 and 6, the signals corresponding to Co^2+^, Cu^2+^, and Zn^2+^ remain close to baseline levels across the tested concentration range. These results confirm that phenanthroline within the PS@Ag + CTAB environment maintains its well-known preference for Fe^2+^ coordination, ensuring that the sensor response is not compromised by the presence of other common transition metals.

**Figure 7:**
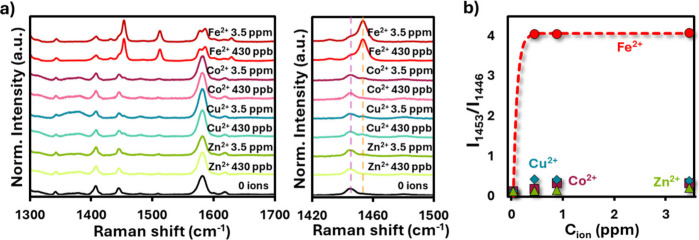
(a) SERRS spectra of PS@Ag + CTAB + Phen in the presence of Fe^2+^, Co^2+^, Cu^2+^, and Zn^2+^ at two different concentrations (*C*_ion_ = 430 ppb and 3.5 ppm) under acidic conditions. The characteristic spectral changes associated with Fe^2+^ complexation (increase at 1453 cm^–1^ and decrease at 1446 cm^–1^) are only observed for Fe^2+^, while other ions show negligible effects. (b) Relative intensity ratio *I*_1453_/*I*_1446_ plotted as a function of ion concentration. A clear Langmuir-type response is obtained exclusively for Fe^2+^, whereas Co^2+^, Cu^2+^, and Zn^2+^ remain close to baseline, confirming the high selectivity of the phenanthroline-based sensor.

To evaluate the performance of the dual-sensing system under more realistic conditions, a mixed sample containing Fe^2+^, Co^2+^, Cu^2+^, and Zn^2+^ (100 ppb each, Milli-Q water, pH ≈ 6) was analyzed.^56^ The SERRS spectrum of the sample (Figure 8) clearly shows both regions of interest: the 1453/1446 cm^–1^ doublet associated with Fe^2+^ complexation by Phen, and the 1587 cm^–1^ band of MBA, which shifts with pH. By deconvoluting the MBA-associated peak at 1586.26 cm^–1^, the pH of the solution was calculated as 5.86, in excellent agreement with the expected value. Using this pH value, the corresponding calibration curve for Fe^2+^ quantification at pH 6 was applied. The relative intensity ratio of the Fe^2+^ marker bands, *I*_1453_/*I*_1446_, yielded a concentration of ∼0.11 ppm (110 ppb). This result is consistent with the nominal Fe^2+^ concentration in the mixed solution, with an error of only ∼10%. These findings demonstrate that the dual-sensing platform can simultaneously and accurately determine both, pH and Fe^2+^ concentration in complex samples containing multiple competing metal ions. Importantly, the selectivity for Fe^2+^ (Figure 7) ensures that the quantification remains unaffected by the presence of Co^2+^, Cu^2+^, or Zn^2+^, highlighting the robustness of the system for real-world applications.

**Figure 8:**
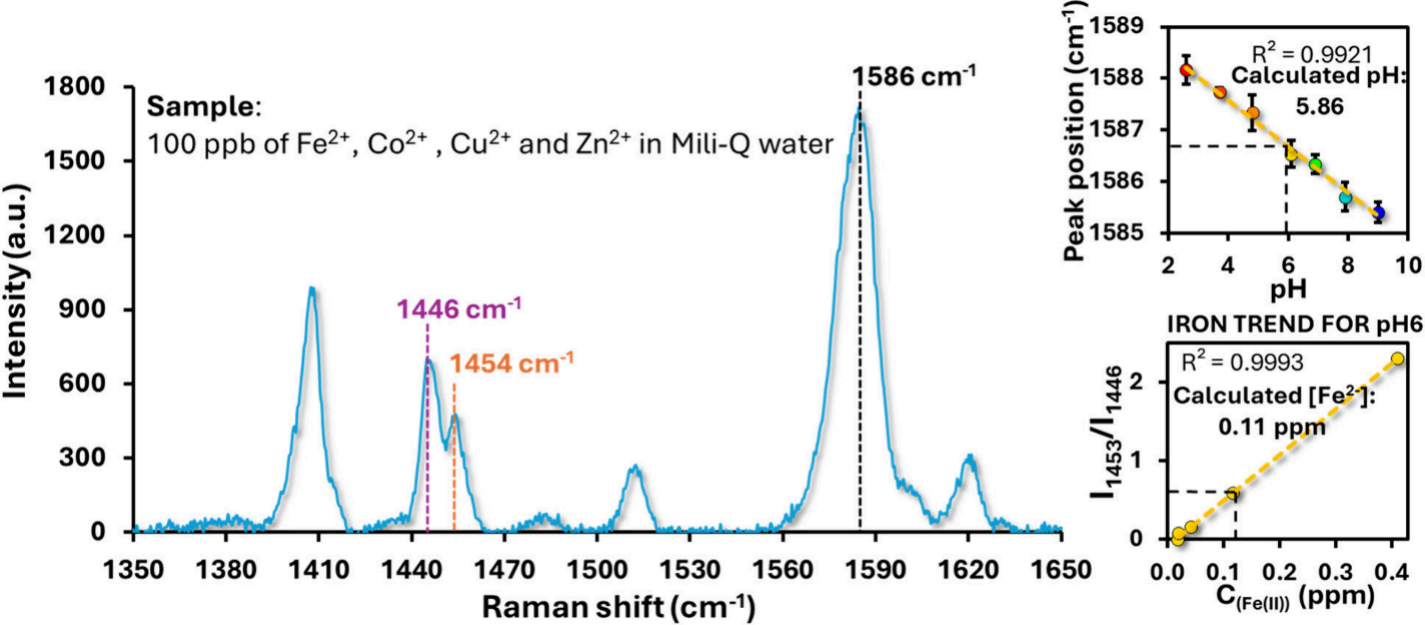
Representative calculation of pH and Fe^2+^ concentration in a mixed-ion solution (100 ppb Fe^2+^, Co^2+^, Cu^2+^, Zn^2+^ in MQ water, pH ≈ 6). Left: SERRS spectrum showing the characteristic Fe^2+^-dependent bands at 1446 and 1454 cm^–1^ and the MBA-associated band at 1586 cm^–1^. Right (top): linear calibration of MBA peak position with pH, used to calculate a sample pH of 5.86. Right (bottom): calibration curve of *I*_1453_/*I*_1446_ versus Fe^2+^ concentration at pH 6, yielding a calculated Fe^2+^ concentration of 0.11 ppm, corresponding to ∼10% error relative to the nominal concentration. R^2^ (coefficient of determination).

## 3 Conclusions

We have developed a facile fabrication strategy for dual-readout SERRS sensors by combining amine-assisted decoration of polystyrene beads with AgNPs, CTAB-mediated entrapment of phenanthroline, and covalent attachment of MBA. This architecture ensures efficient retention of the ion-selective dye in close proximity to plasmonic hotspots without chemical modification, thereby preserving its Fe^2+^-binding capacity. The resulting sensor achieves ultrasensitive Fe^2+^ quantification down to 30 ppb while simultaneously providing a reliable pH readout, effectively overcoming one of the main limitations of optical ion sensing. While demonstrated here for Fe^2+^ as a proof of concept, the platform is designed to be generalizable by substituting phenanthroline with other ion-selective dyes, rather than serving as a universal Fe^2+^ sensor across all pH regimes. Moreover, although demonstrated in aqueous solutions, this methodology can be readily extrapolated to more complex environmental fluids and biological systems, paving the way for robust, selective, and versatile plasmonic sensors for metal ion monitoring in real-world conditions.

This dual sensing (Fe^2+^ or other analytes, together with pH) may have high relevance for intracellular sensing. The pH varies drastically inside cells, from highly acidic endosomes/lysosomes to the almost neutral cytosol.^57^ In a potential scenario, a particle responsive to an analyte would be added to cells. After exposure in the almost neutral cell medium, this particle would be endocytosed, leading to a highly acidic local environment.^58^ After possible endosomal escape, the particle would again be located in the neutral pH of the cytosol.^59^ If the time-dependent local concentration of the analyte should be measured, one needs to take into account the continuous changes in pH, as they interfere with the readout. By measuring the pH in parallel, as done in this study, the analyte readout can be pH-corrected, potentially allowing for in situ monitoring of analyte concentrations in variable environments.

## 4 Experimental Section

### 4.1 Chemicals

Aliphatic amine polystyrene beads (PS beads, 0.31 ± 0.011 μm diameter, 2%) were purchased from ThermoFisher, with an amine charge titration of 224.3 μEq/g and a carboxyl charge titration of 16.9 μEq/g. 1,10-Phenanthroline monohydrate (>99%, Phen) and 4-mercaptophenol (99%, MPH) were also obtained from ThermoFisher. Silver nitrate (>99%, AgNO_3_) was purchased from Honeywell. l-ascorbic acid (>99%, AA), sodium citrate tribasic dihydrate (>99%, C_6_H_5_Na_3_O_7_), iron­(III) nitrate nonahydrate (>98%, Fe­(NO_3_)_3_), hexadecyltrimethylammonium bromide (>96%, CTAB), 4-mercaptobenzoic acid (90%, MBA), 4-Mercaptophenol (97%, MPH), iron­(II) sulfate heptahydrate (>99%, FeSO_4_·7H_2_O), copper­(II) sulfate (CuSO_4_), cobalt­(II) nitrate hexahydrate (>98%, Co­(NO_3_)_2_), zinc (>98%), acetic acid (>99%, CH_3_COOH), sodium acetate (>99%, CH_3_COONa), potassium phosphate monobasic (>99%, KH_2_PO_4_), sodium carbonate (>99%, Na_2_CO_3_), and sodium bicarbonate (99.5–100%, NaHCO_3_) were purchased from Sigma-Aldrich. Sodium hydroxide (>98%, NaOH), and hydrochloric acid (37%, HCl) were purchased from Roth, and absolute ethanol (>99.8%, EtOH) from VWR. All chemicals were used as received without further purification. Milli-Q water (18 MΩ cm^–1^) was used for all aqueous solutions. All glassware was cleaned with freshly prepared aqua regia, rinsed thoroughly with deionized water, and washed with ethanol prior to use.

### 4.2 Instrumentation

Transmission electron microscopy (TEM, JEOL JEM-1011) at 100 kV and scanning electron microscopy (SEM, Hitachi Regulus 8220) at 10 kV were used to characterize nanoparticle size and morphology. Samples were prepared by drop-casting colloidal suspensions onto carbon–Formvar-coated copper grids (200 mesh). UV–vis absorption spectra were acquired on a Cary 60 spectrophotometer (Agilent). A Thermo Scientific Orion Star A111 pH meter was used to measure buffer pH. Raman/SERS/SERRS experiments were conducted with a Horiba LabRAM HR Evolution dispersive spectrometer coupled to an Olympus BX-FM confocal microscope. Excitation was provided by a frequency-doubled Nd:YAG laser (532 nm) and, when indicated, a 785 nm laser. A grating of 1800 gr/mm and a Syncerity 2D CCD detector were used.

### 4.3 Synthesis of Silver Nanoparticles (AgNPs)

AgNPs (∼15 nm) were prepared following a modified protocol.^36^ Briefly, 250 mL of Milli-Q water was heated to boiling, after which 250 μL of freshly prepared 0.1 M ascorbic acid and 1.5 mL of 0.1 M sodium citrate were added under vigorous stirring. After 1 min, a mixture of 496 μL of 0.1 M AgNO_3_ and 198.4 μL of 0.01 M Fe­(NO_3_)_3_ (preincubated for 5 min) was introduced. The solution underwent a rapid color change (colorless → black → orange → yellow), indicating nanoparticle formation. The reaction was maintained at boiling for 1 h, then cooled and stored in the dark.

### 4.4 Assembly of AgNPs on PS Beads

To obtain dense AgNP coverage, the stoichiometric amount of AgNPs required to fully cover 300 nm PS beads was calculated by considering the surface area of the beads and the footprint (area) of the AgNPs. The result was then multiplied by five to ensure an excess. PS beads (1 mg/mL) were mixed with AgNPs (∼1.5 × 10^12^ particles/mL) and shaken overnight. The next day, PS@Ag were purified by three centrifugation cycles (6000 rpm, 10 min centrifugation, removal of supernatant, redispersion in Mili-Q water) and redispersed in Milli-Q water to a final bead concentration of 0.5 mg/mL.

### 4.5 CTAB Coating and Phen Adsorption

Equal volumes of PS@Ag beads (0.5 mg/mL) and CTAB (1 mM) were mixed under sonication and incubated for 15 min. The resulting PS@Ag + CTAB were centrifuged (5000 rpm, 5 min), the supernatant was discarded, and redispersed in Milli-Q water to a final bead concentration of 0.05 mg/mL. For phenanthroline entrapment, 10 μL of Phen at different concentrations (10^–2^ – 10^–8^ M, in EtOH) was added to 990 μL of PS@Ag + CTAB (0.05 mg/mL) and incubated for ≥ 2 h. For kinetic studies, a final Phen concentration of 1 mM was used. For Fe^2+^ sensing experiments, PS@Ag@CTAB (0.05 mg/mL) were incubated with Phen (final Phen concentration = 10 μM) for 3 h, centrifuged (5000 rpm, 5 min) with discarding of the supernatant, and redispersed in Milli-Q water or pH buffer with the corresponding iron concentration (as described in the sample preparation section) to a final bead concentration of 0.05 mg/mL.

### 4.6 MBA Functionalization

For pH sensing, PS@Ag (0.05 mg/mL) were incubated with MBA and MPH (5 μL, 1 mM in EtOH per mL of a prepared suspension of MPH:MBA (1:10)) overnight.

Note that MPH was initially added because we thought it could be useful to have a pH sensor capable of sensing pH from 3 to 12. However, since MPH was added in a very low concentration, no signal was observed. At the same time, using only MBA, we can sense the pH range of interest 3–7. For this reason, in the article we refer only to MBA, the experiments would have led to the same result without having added MPH.

After incubation, PS@Ag + MBA were centrifuged (6000 rpm, 10 min) to form a precipitate with discarding of the supernatant and redispersed in buffers of pH 3–9. For dual-readout experiments, PS@Ag + Phen and PS@Ag + MBA were prepared separately and mixed 1:1 before measurements.

### 4.7 Buffer Preparation

Seven buffers covering pH 3–9 were prepared using standard mixtures: pH 3–6: acetic acid/sodium acetate; pH 7–8: KH_2_PO_4_/NaOH; pH 9: Na_2_CO_3_/NaHCO_3_.

Volumes were adjusted as listed in Table 1, Table 2, and Table 3. Buffers were prepared with Milli-Q water and fine-adjusted with 0.1 M NaOH or HCl. pH was confirmed with a calibrated pH meter.

**Table 1:** Adjusted Volumes for the Preparation of pH Buffer at pH 3, 4, 5, and 6

pH	C_2_H_4_O_2_ (0.1 M)	CH_3_COONa (0.1 M)
3	491.15 mL	8.85 mL
4	423.5 mL	76.5 mL
5	178.5 mL	321.5 mL
6	12.6 mL	473.9 mL

**Table 2:** Adjusted Volumes for the Preparation of pH Buffer at pH 7 and 8

pH	KH_2_PO_4_ (0.1 M)	NaOH (0.1 M)
7	300 mL	174.6 mL
8	300 mL	280.2 mL

**Table 3:** Adjusted Volumes for the Preparation of pH Buffer at pH 9

pH	Na_2_CO_3_ (0.1 M)	NaHCO_3_ (0.1 M)
9	50 mL	450 mL

### 4.8 Sample Preparation and Measurements

Samples were measured in liquid using quartz cuvettes and a UV–Vis–NIR macro cuvette holder. Unless otherwise noted, excitation was performed at 532 nm (13.2 mW, 1800 gr/mm grating). Acquisition time and accumulations were adapted per experiment.

#### 4.8.1 Phen Characterization

One mL of Phen (3 mM in Milli-Q) was measured at 532 and 785 nm. Ferroin was prepared by mixing 30 μL of 0.1 M Phen with 970 μL of 1 mM Fe^2+^. For SERS characterization 1 mL of PS@Ag (0.05 mg/mL) with Phen at 10 μM and Fe2+ at 300 μM was measured under 532 nm (13.2 mW, 1800 gr/mm grating) and 785 nm (14.4 mW, 1800 gr/mm grating) excitation.

#### 4.8.2 Phen Adsorption Isotherm/Kinetics

PS@Ag + CTAB (0.05 mg/mL) incubated with Phen at varying concentrations or 1 mM for time studies. Spectra collected from 30 s to 4 h after addition.

#### 4.8.3 Fe^2+^ Sensing

FeSO_4_ was added to PS@Ag + Phen suspensions (0 – 3 M stock solutions, final concentrations from ppb to ppm). After 1 h incubation, spectra were recorded. Time-resolved experiments monitored spectra every 10 s after Fe^2+^ addition.

#### 4.8.4 pH Sensing

PS@Ag-MBA suspensions were measured across buffers (pH 3 – 9).

#### 4.8.5 Dual-Readout Sensing

0.5 mL of PS@Ag + Phen and 0.5 mL of PS@Ag + MBA were combined in buffers containing different Fe^2+^ concentrations and incubated for 40 min before measurements.

#### 4.8.6 Ion Interference Study

PS@Ag sensors were exposed to Fe^2+^, Co^2+^, Cu^2+^, or Zn^2+^ (0 – 6 mM) at pH 4 or 7. After 1 h incubation, spectra were recorded.

## Supplementary Material



## Notes

Additional Raman, SERS, SERRS, and UV–vis spectral information for sensor construction and characterization (PDF)

## ABBREVIATIONS

SERRS: surface-enhanced resonance Raman scattering
CTAB: cetrimonium bromide
MBA: mercaptobenzoic acid
AAS: atomic absorption spectroscopy
AES: atomic emission spectroscopy
ICP-MS: inductively coupled plasma mass spectrometry
SERS: surface-enhanced Raman scattering
MOF: metal–organic framework
PS: polystyrene beads
NP: nanoparticles
Phen: Phenanthroline
TEM: transmission electron microscopy
LSPR: localized surface plasmon resonance
SEM: scanning electron microscopy
MLCT: metal-to-ligand charge transfer
UV: ultraviolet
LOD: limit of detection
MPH: mercaptophenol
NIR: near-infrared
