# Supplementary material for: One-Shot SERRS Detection of Iron and Acidity in Aqueous Systems
Source: ACS Appl Mater Interfaces. 2026 Jan 14;18(3):5751–61. doi: 10.1021/acsami.5c22602 (PMC12862772; doi:10.1021/acsami.5c22602)
Supplement: Supplementary file 1 [file am5c22602_si_001.pdf]

Supporting Information

One-Shot SERRS Detection of Iron and Acidity in Aqueous Systems

Irene Calderón González‡[1], Robbert Schuett‡[2], Gerwin Chilla[2], Florian Schulz[2], Zhiming Wang[3,4] Wolfgang J. Parak[2], Ramon A. Alvarez-Puebla*[1,5]*

[1]Department of Physical and Inorganic Chemistry, Universitat Rovira i Virgili, Tarragona, Spain.

[2]Fachbereich Physik, Universität Hamburg, Hamburg, Germany

[3]School of Physics, University of Electronic Science and Technology of China, Chengdu, 611731, China

[4]Shimmer Center, Tianfu Jiangxi Laboratory, Chengdu 641419, People's Republic of China

[5]ICREA – Institució Catalana de Recerca i Estudis Avançats, Barcelona, Spain.

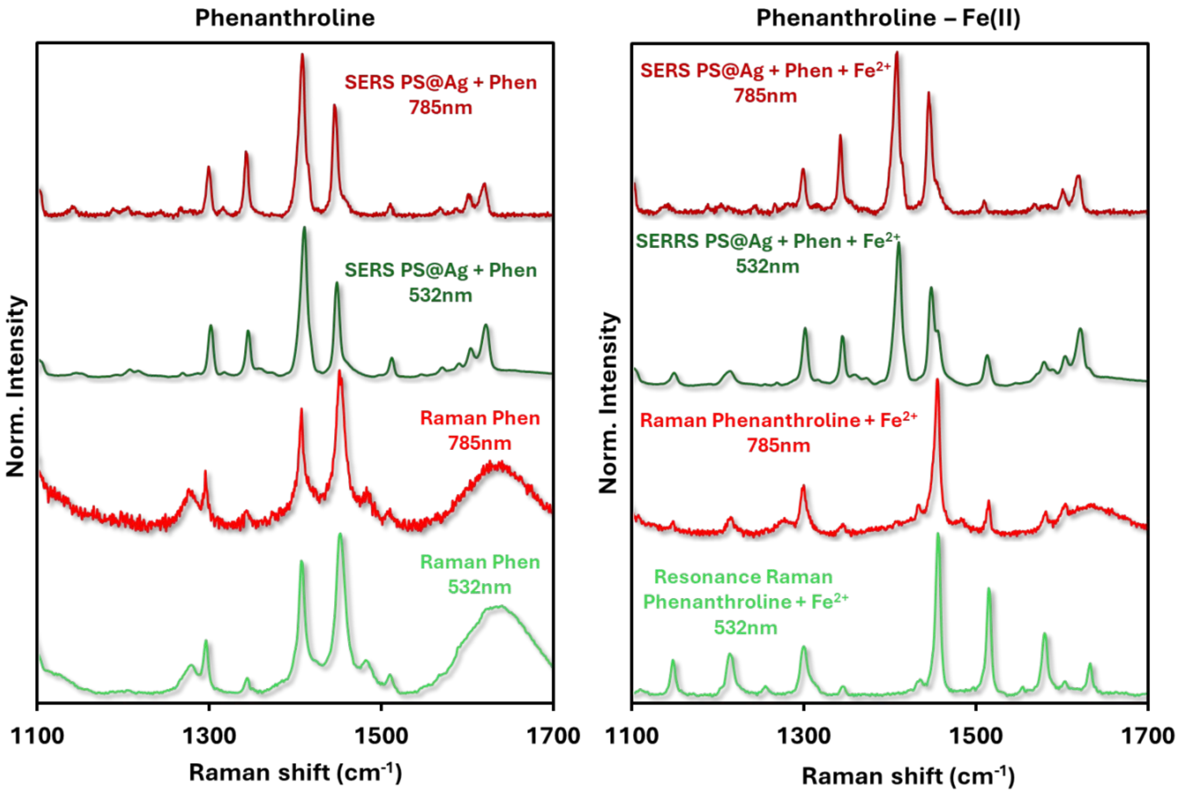

Figure S1. Raman, resonance Raman SERS, and SERRS spectra of free Phen and its Fe(II) complex (ferroin) under 785 nm (red) and 532 nm (green) excitation, showing the normalized intensities of the respective Raman shifts.

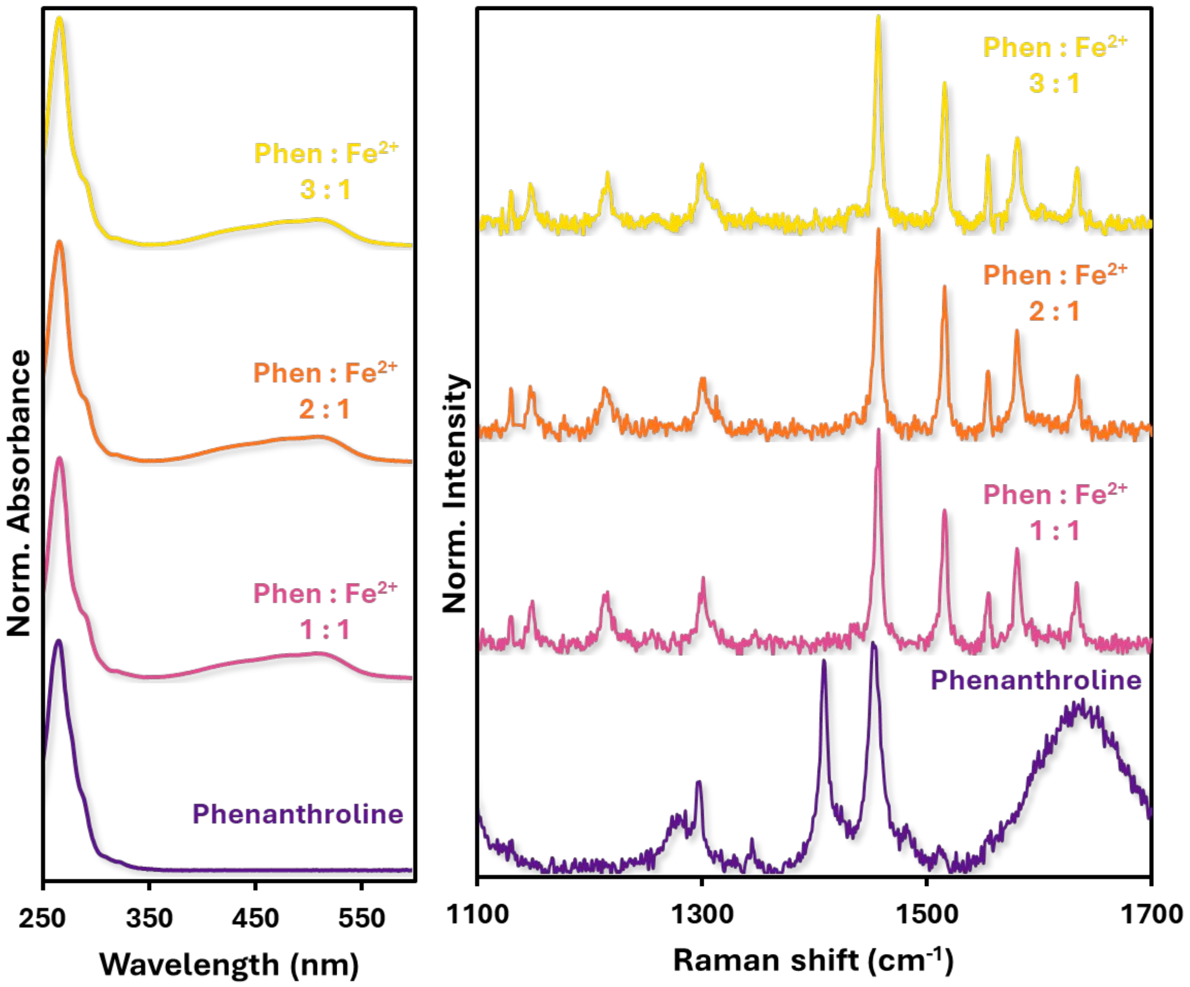

Figure S2. UV–vis absorption spectra (left) and Raman spectra (right) of phenanthroline and its Fe(II) complexes at different ligand-to-metal ratios (1:1, 2:1, and 3:1). The absorption profiles are dominated by the π→π* and n→π* transitions of Phen and by the MLCT band of ferroin in the visible region, which remains essentially unchanged across stoichiometries. Raman spectra similarly display consistent vibrational features, confirming that the Fe(II) concentration can be quantified independently of the coordination number.

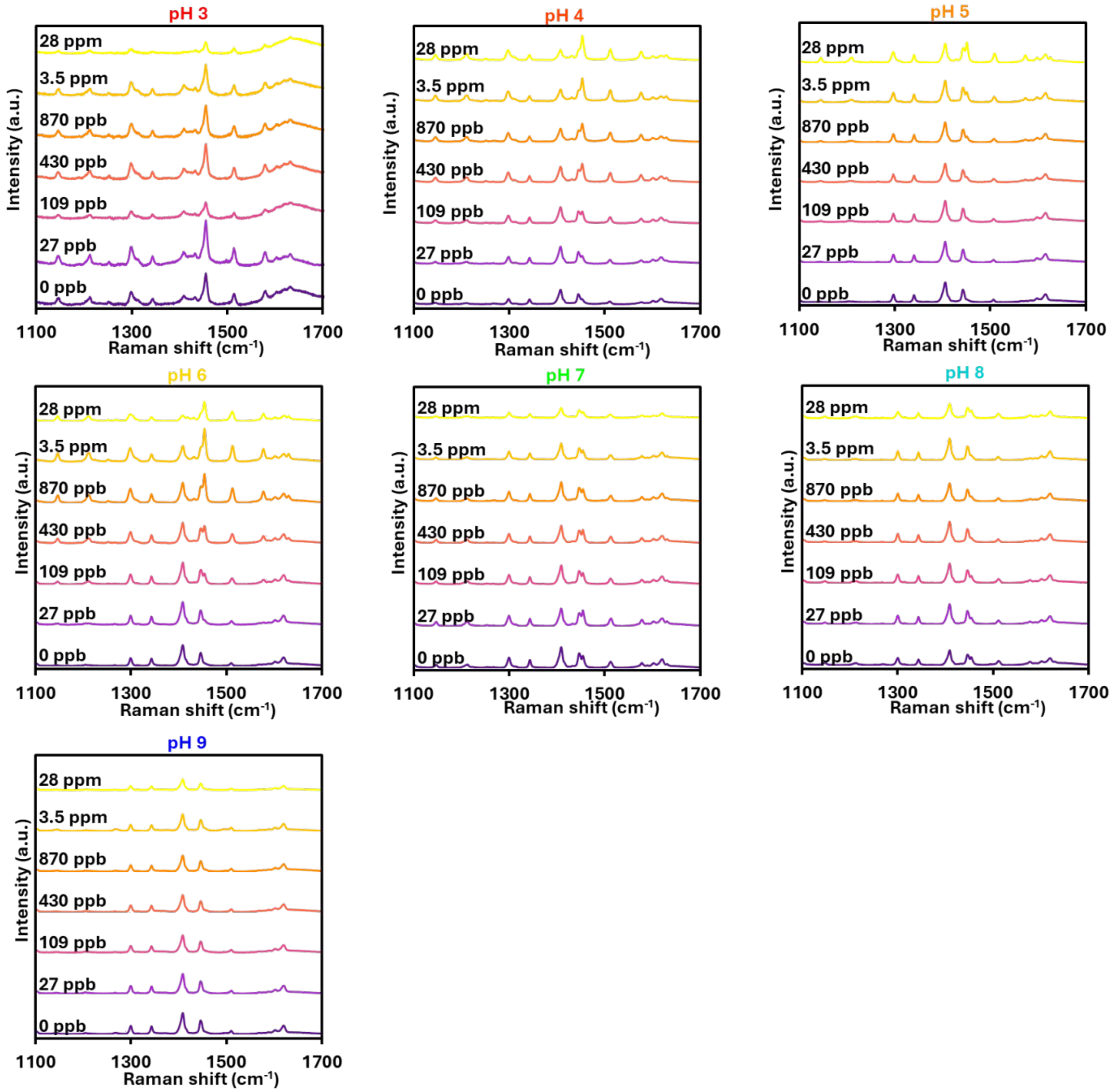

Figure S3. Complete SERRS spectra of PS@Ag + CTAB + Phen in the presence of increasing Fe(II) concentrations at different pH values (3 – 9). Spectral changes corresponding to ferroin formation (1453 cm^{-1} increase, 1446 cm^{-1} decrease) are clearly observed under acidic conditions (pH 3–6), while no significant variation occurs at neutral or basic pH (pH 7 – 9).

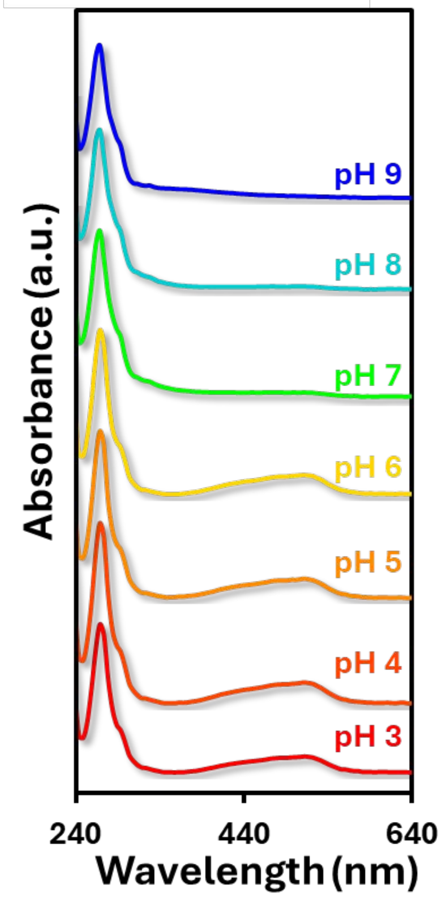

Figure S4. UV–vis absorption spectra of phenanthroline - Fe(II) complexes at different pH values. The absorption profiles are dominated by the π→π* and n→π* transitions of phenanthroline across all pH conditions. The MLCT band of ferroin in the visible region is prominent only under acid conditions (pH 3 - 6), confirming the absence of complex formation at pH values above 7.

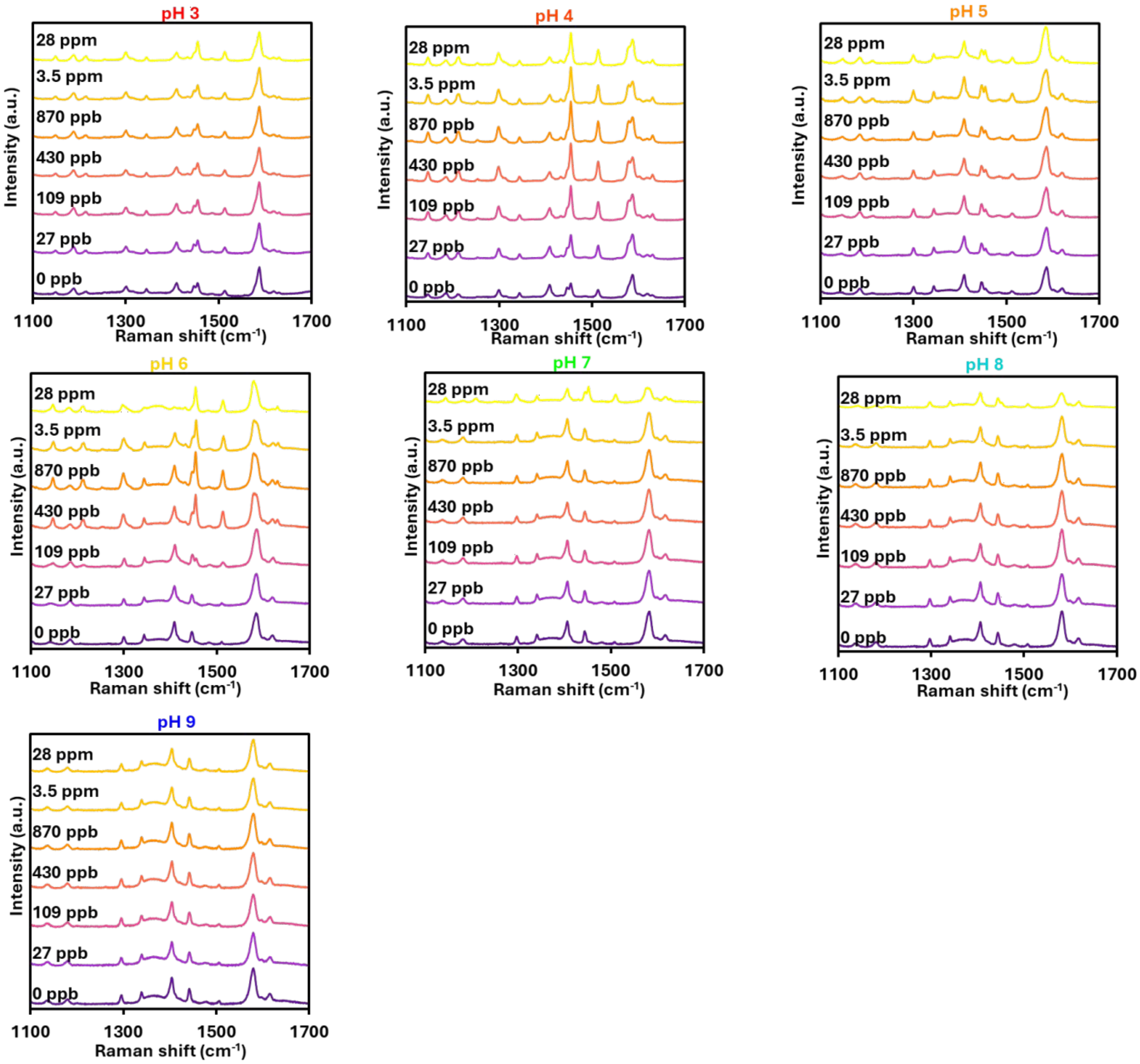

Figure S5. Complete SERRS spectra of PS@Ag + CTAB + Phen mixed with PS@Ag + MBA at different Fe^{2+} concentrations (0 – 28 ppm) and pH values (3 – 9). The combined spectra confirm the simultaneous presence of Phen and MBA vibrational markers across the full pH range.

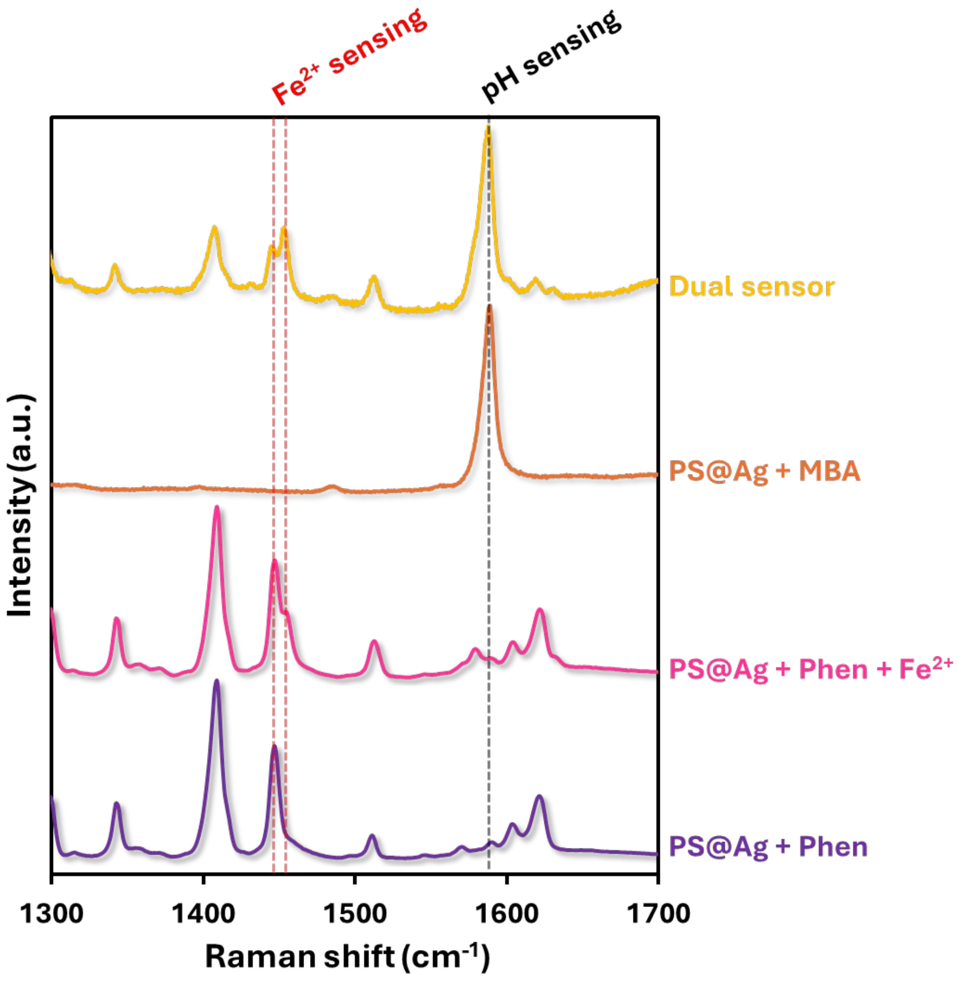

Figure S6. SERRS spectra of PS@Ag + CTAB + Phen, PS@Ag + CTAB + Phen + Fe^{2+}, PS@Ag + CTAB + MBA, and a mix of both sensors. The combined spectra confirm the simultaneous presence of Phen and MBA vibrational markers, and single spectra demonstrate non-overlapping of the selected bands.

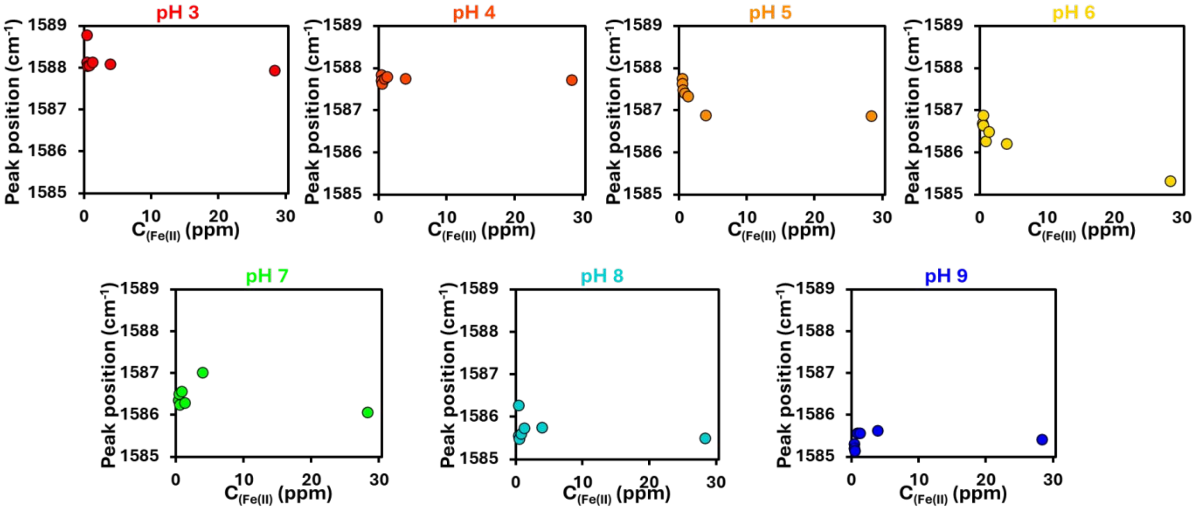

Figure S7. Peak position of the MBA ring stretching vibration (~1587 cm^{-1}) plotted as a function of Fe^{2+} concentration at different pH values (3 – 9). No dependence on Fe^{2+} concentration is observed, confirming that the MBA marker provides a selective and independent pH readout in the multiplexed sensing system.
